# Heart Rate Variability Responses in Children With Medical Complexity Across Sensory Domains

**DOI:** 10.1155/ijpe/5803653

**Published:** 2025-09-15

**Authors:** Cindy Dodds, Brooke Mulrenin, Carrie Cormack, Kimberly Kascak, Nia Mensah, Marianne Gellert-Jones, Keri Heilman, Mat Gregoski, Everette Keller, Jonathan Beall

**Affiliations:** ^1^College of Health Professions, Medical University of South Carolina, Charleston, South Carolina, USA; ^2^College of Nursing, Medical University of South Carolina, Charleston, South Carolina, USA; ^3^Office of Interprofessional Initiatives, Medical University of South Carolina, Charleston, South Carolina, USA; ^4^Biokinesiology and Physical Therapy Department, University of Southern California, Los Angeles, California, USA; ^5^HMS School, Philadelphia, Pennsylvania, USA; ^6^Department of Psychiatry, University of North Carolina, Chapel Hill, North Carolina, USA; ^7^Department of Public Health Sciences, Medical University of South Carolina, Charleston, South Carolina, USA

**Keywords:** autonomic dysfunction, autonomic nervous system, cerebral palsy, children with medical complexity, heat rate variability, parasympathetic regulation, respiratory sinus arrhythmia

## Abstract

**Purpose:** Heart rate variability (HRV) is a physiologic marker of autonomic nervous system (ANS) health. Because of multisystem impairments, children with medical complexity (CMC) can be challenging to evaluate and assess. They commonly display ANS dysfunction and subsequently reduced HRV. One purpose of this study was to report respiratory sinus arrhythmia (RSA) values derived from HRV, which may provide a reference and a future method to assess CMC and related treatments.

**Methods:** Then, 36 CMC between the ages of 5–21 years were assessed within school-based settings. All children demonstrated severe brain impairment, functional limitations, participatory restrictions in life, and high healthcare needs and utilization. A subset of 20 children with cerebral palsy (CP) was identified within the study sample. Each child was assessed by the same assessor for 5 days within a 10-day period. Using Firstbeat Bodyguard 2 monitors, cardiac data were collected prior to and during the administration of the newly developed Pediatric Assessment of Sensory Motor Awareness. CardioEdit and CardioBatch software were used to edit cardiac data and calculate RSA values. Descriptive, repeated measures, and modeling statistics were used to analyze data.

**Results:** Descriptive RSA values for baseline and across five sensory domains are reported for the total CMC group and its subsets of children with CP and non-CP. Significant differences in RSA values were noted for the gustatory domain as compared to baseline and other sensory domains. Significant differences from statistical modeling were demonstrated between the CP and non-CP groups.

**Conclusions and Recommendations for Clinical Practice:** This study's HRV findings provide preliminary reference RSA values in CMC. Then, 20 children considered to be CMC with primary diagnoses of CP experienced less autonomic dysfunction than other CMC diagnostic groups at baseline and across the five sensory domains. The gustatory domain significantly reduced RSA. Modeling across days indicated that CMC experience a learning effect by Day 3 or 4. The measurement of HRV may provide a method to assess health and behavioral responses in CMC and associated treatments. Replication of these study findings will be necessary.

## 1. Introduction and Purpose

In the United States, 13%–18% of children have special health care needs, with 63% requiring specialty medical care [1]. Children with medical complexity (CMC) are a small subgroup of this larger population and are estimated to represent about 0.7% of all children [2]. CMC demonstrate heterogeneity [3] and multisystem impairments that result in considerable functional activity limitations (e.g., standing, reaching, and talking) and participatory restrictions (e.g., recreation and leisure activities) [2, 4, 5]. Diagnostically, CMC present with a variety of primary and co-occurring health conditions. Common primary diagnoses may include cerebral palsy (CP), genetic diseases, traumatic brain injury, or hypoxic encephalopathy. Although CMC are a small subgroup, because of frequent and extensive hospitalizations, primary and specialty physician visits, pharmacology, medical equipment, rehabilitation, and care coordination, healthcare utilization is one of the highest [2, 6].

Because of the multiple impairments and activity limitations, caregivers [7] and practitioners [8, 9] may find it challenging to assess CMC's health and behavioral responses during evaluation and treatment. One possible solution to the problem of evaluating status and responses may be to include a physiological measurement, such as heart rate variability (HRV). HRV is an index of the state of the autonomic nervous system (ANS), and respiratory sinus arrhythmia (RSA), the primary component of high-frequency HRV, specifically reflects the parasympathetic effect of the myelinated vagus nerve on the heart. A reduction in RSA indicates parasympathetic withdrawal to the heart, thereby supporting a sympathetic response (i.e., fight or flight), while an increase in RSA indicates greater parasympathetic regulation of the heart, which supports states of rest and digest or homeostasis and repair. As internal and environmental contextual demands change, a healthy ANS shifts between sympathetic and parasympathetic states when necessary.

Today outside of traditional 12-lead electrocardiogram (ECG), there are a variety of methods for collecting and evaluating HRV, including wearable devices that use ECG to record the time between R-to-R peaks. These wearable devices also are associated with various software programs which can objectively calculate frequency and time domains, including RSA derived from HRV. These values can then be used to interpret the dynamic state of the ANS [10, 11].

Chronic conditions such as brain damage, poor cardiovascular conditioning, and pain often lead to decreases in HRV that infer chronic ANS dysfunction and poorer long-term health in a variety of diagnostic categories, including CMC [12–14]. HRV has been used to describe the autonomic dysfunction of children with CP [12, 15] and autism [16, 17] with both groups having demonstrated lower RSA values. With specific regard to children with autism [17] and Fragile X syndrome [18], the polyvagal theory [10, 11] has provided a neurophysiological mechanistic underpinning as to how sensory impairments alter RSA values. More broadly conceptualized in Porges' polyvagal theory [10, 11], the purpose of this paper is to describe parasympathetic regulation as defined by RSA values in CMC across a baseline period and five sensory domains to better inform rehabilitation assessment and treatment.

## 2. Methods

### 2.1. Human Subject Protection

The Institutional Review Board of the Medical University of South Carolina approved this study. Parental informed consent and Health Insurance Portability and Accountability Act (HIPAA) privacy documentation were obtained from parents. Secondary to motoric and cognitive impairments, the study participants' assents were not sought.

### 2.2. Study Design

The study is a prospective descriptive cohort study.

### 2.3. Participants

Then, 36 medically stable children between the ages of 5–21 years with physician-reported severe brain damage participated and were considered to be representative of CMC. Children were confirmed by physician or nurse to be medically stable, which was defined as the child's medical treatment having reached a point where life-threatening injuries and diseases had been brought under control. Then, 20 of the CMC had a primary health condition of CP. The remaining 16 children were diagnostically heterogeneous with primary health conditions that included traumatic brain injury, hypoxic encephalopathy, and genetic diseases. Children greater than 5 years of age were included because HRV stabilizes following early postnatal life [19]. Children up to 21 years were included because they are considered pediatric with respect to receiving services from children's hospitals, pediatric healthcare providers, and public school systems. All children were dependent and required caregiver assistance for mobility, self-care, and communication. For the children with CP, 19 could be considered level V on the Gross Motor Function Classification System (GMFCS) [20] with one child being a GMFCS level IV. Along with demographic information for all study participants, the Manual Ability (MACS) [21], Communication Function (CFCS) [22], and Eating and Drinking Abilities (EDACS) [23] Classification System descriptions can be found for children with CP in 1.

### 2.4. Settings

Pattison's Academy in Charleston, South Carolina; iHope in New York, New York; HMS School in Philadelphia, Pennsylvania; and two children's homes (home-bound students) in Charleston, South Carolina served as sites for the Pediatric Assessment of Sensory Motor Awareness (PASMA) administration and all associated data collection, including HRV. The assessment administration took place at a location chosen by parents/legal guardians as long as a caregiver familiar with the child was present, such as a personal care aide, nurse, or teacher.

### 2.5. Outcome Measurement Tools

#### 2.5.1. PASMA

The PASMA was developed by a collaborative, interprofessional (IP) team of the Medical University of South Carolina faculty and graduate student members from the Colleges of Health Professions and Nursing as well as colleagues from a community partner, Pattison's Academy. The PASMA was modeled after the validated adult version of the Sensory Modality Assessment and Rehabilitation Technique (SMART) which was developed as a diagnostic neurobehavioral tool to comprehensively assess minimally responsive adults [24, 25]. Although the Adult SMART served as the foundation for the PASMA, evidence-based literature, body structure and function knowledge, expert opinion, and clinical expertise further informed the development of the PASMA. The PASMA consists of a 5-min observation period during which the assessor observed and documented the child's common motor behaviors during a quiet and alert state. This observational period is followed by an evaluation of sensory motor responses across 28 items structured across five sensory domains (i.e., olfactory, visual, auditory, gustatory, and tactile). See Table 1 for an item example across each sensory domain. Four of the IP team members were formally trained and certified in the administration of the Adult SMART, which included establishing reliability. Three of these individuals carried out all study-related PASMAs. The PASMA was designed to be administered five times over 10 days. Prior to each testing, the child's initial heart rate, blood pressure, respiratory rate, awareness level (i.e., deep sleep, light sleep, drowsy, quiet awake, active awake, and crying), and test location (i.e., home, school, hospital, office, and rehab center) and positioning (i.e., bed, wheelchair, and other) were documented. Ideal time of day (i.e., morning and afternoon) may vary for individual CMC, and as such, if three of the assessments took place in the morning, then two occurred in the afternoon or vice versa. This was carried out to minimize the impact of time of day on children's performances and related findings. Electronic tablet technology was used to organize PASMA items and capture item responses and scores. This manuscript highlights descriptive RSA gathered during the construct and concurrent validation of the PASMA. Validation findings for the PASMA will be reported in a second manuscript.

#### 2.5.2. Firstbeat Bodyguard 2

The Firstbeat Bodyguard 2 (Firstbeat Technologies Ltd, Jyväskylä, Finland) is a portable, wearable beat-to-beat heart rate monitor that was designed to measure HRV and physical activity. Its accuracy in measuring the beat-to-beat or R-to-R peak interval was tested against a clinical ECG in 19 healthy participants during rest and physical activity. The Firstbeat Bodyguard 2 accurately measures 99.95% of the heartbeats compared to standard ECG [26]. CardioEdit and CardioBatch software (CardioEdit and CardioBatch software 2007 Brain-Body Center, University of Illinois at Chicago, Chicago, IL) and Firstbeat Analytics (2023 Firstbeat Analytic, https://www.Firstbeatanalytics.com/en/, Finland) were used to edit beat-to-beat data, calculate RSA, and calculate root mean square of successive differences (RMSSD), respectively.

Two methods of editing and analyzing HRV were used. The majority of data was edited and analyzed using CardioEdit and CardioBatch software (Brain-Body Center, University of Illinois at Chicago, Chicago, IL) which edited R-R intervals (i.e., heart period) and calculated RSA. Editing consisted of visual inspection of the R-R peaks and manual editing of arrhythmias, missed beats, and/or artifacts using integer arithmetic (i.e., addition of multiple detections of spurious R-peaks, division when missed beats were present, and combination of addition/division to edit more complex arrhythmias and artifacts). RSA was derived from the edited heart period via CardioBatch, which applied the Porges method [27] to calculate RSA. The Porges method uses a time-frequency algorithm to quantify the amplitude of RSA with age-specific parameters, sensitive to the maturational shifts in the frequency of spontaneous breathing. Steps included timing the R-R intervals to the nearest millisecond to produce a time series of sequential heart periods; resampling the heart periods into 250-ms intervals to produce time-based data; detrending the time-based series using a 51-point cubic moving polynomial [28] to create a smoothed template, which is then subtracted from the original time-based series to generate a detrended residual series; using a bandpass filter to detrend the series to restrict the variance in the heart period pattern associated with spontaneous breathing (i.e., 0.12–1.00 Hz to include the range of spontaneous breathing for the wide age range of participants); calculating the natural logarithm of the variance of the bandpassed time series as the measure of the amplitude of RSA [29]. Available data from each participant were analyzed in 30-s epochs to compute a grand average over the entire recording. All editing and analyses were accomplished by reliable users of the software. The second source of editing and analyses took place with Firstbeat Analytics (2018 Firstbeat Analytic, https://www.Firstbeatanalytics.com/en/, Finland). It too uses beat-to-beat data and calculates the RMSSD for this study. ECG data from the Firstbeat BodyGuard 2 was uploaded using the Firstbeat Uploader and saved on the server. These files were then shared with a reliable Firstbeat consultant with access to the proprietary Firstbeat Analytic software. He uploaded files into the software, visually inspected them, edited artifacts, and produced data analysis reports from which RMSSD values were provided and recorded in the study database.

### 2.6. Procedures

After obtaining informed consent from parents and legal guardians, children's healthcare practitioners (i.e., physician, physician assistant, nurse practitioner, and registered nurse in school or home health setting) were approached by the principal investigator (PI) to provide written documentation confirming the participant's medical stability and presence of severe brain impairment. Following confirmation, participants were assigned an identification number to avoid confidentiality breaches and limit bias during data analyses. Parents, legal guardians, and the PASMA administrator identified five dates within 10 calendar weekdays and appropriate locations (i.e., Pattison's Academy, iHope, HMS School, and children's homes) for the administration of study PASMAs. Five PASMAs were completed within 10 days for each participant.

Three developers of the PASMA (physical therapist (PI), nurse practitioner, and certified special educator), who were also certified administrators of the Adult SMART, carried out all PASMA administrations and data collection on study participants. Each administrator had more than 15 years of experience serving CMC. These PASMA administrators were not blinded, and interrater reliability was not established between raters; however, reliability for the Adult SMART administration had been achieved and certified for all raters prior to this study's implementation.

All administrators were trained and able to properly apply and remove the Firstbeat monitors as well as transfer HRV data from the monitors to the secure shared server. During each PASMA administration, Firstbeat monitor electrodes were placed below the right sternum and left lateral rib region. Each PASMA administration was video recorded to allow timing synchronization of HRV across the baseline and each sensory domain time period. The sequence of test administration was as follows: 5-min baseline observational period, olfactory, visual, auditory, gustatory, and tactile domain test items. A short rest period occurred between each sensory domain to allow administrators to record PASMA scores. These scores were recorded on iPads and stored within the REDCap electronic data capture system [30, 31]. Because of the large file size, HRV data could not be stored within the REDCap system and the decision to use a secure shared server was made.

### 2.7. Data and Statistical Analyses

Our primary outcome was RSA derived from HRV and was calculated at baseline and across the domains of olfactory, visual, auditory, gustatory, and tactile. Three trained undergraduate and graduate students reviewed study video recordings to document the beginning and ending times of baseline and each of the sensory domains. The PI and doctoral-level student were trained and demonstrated reliability in the use of CardioEdit to edit artifacts and CardioBatch to calculate RSA values for baseline and sensory domains. Calculations for total RMSSD values from HRV files were provided by an external consultant with expertise using Firstbeat Analytic (2023 Firstbeat Analytic, https://www.Firstbeatanalytics.com/en/, Finland).

A repeated measures multivariate joint Bayesian hierarchical model (BHM) was constructed to assess for associations between domain test items and RSA values. The BHM examines the trends both across the domains of interest and a set of relevant predictors including a linear time trend, a quadratic time trend, and an effect for CP [32]. In addition, BHMs are capable of implementing random effects to address the extra noise and heterogeneity in the trajectories within each modality across each visit as well as adjust for the correlation that commonly exists when there are repeated outcome observations per participant. See Appendix 1.

Of the 180 potential HRV files collected, approximately 31.85% of the possible RSA values were missing across the 36 participants. Missing RSA values were primarily a result of instrumental (e.g., Firstbeat monitor electrodes dropped off and Firstbeat monitor batteries lost charge) and environmental (e.g., bracing or wheelchair postural supports interfered with Firstbeat monitor's recording) systematic error associated with HRV data collection. Given this expectation for the cause of missing data, we assumed the data were missing at random and proceeded with multiple imputation to correct for the missing data. Our approach assumed the probability of the RSA outcomes being missing is conditionally independent of the unobserved data. Under our Bayesian paradigm, this was addressed by allowing RSA outcome values to be predicted from the model. The predictive distribution automatically imputed the missing values during the Markov Chain Monte Carlo (MCMC) sampling, and data augmentation was implemented to obtain appropriate posterior estimates [26].

## 3. Results

Participant characteristics are described in Table 2. Participants were stratified by CP or non-CP. Testing parameters documented that 92 of sessions took place in the morning and 88 in the afternoon; 14 assessments occurred in homes and 166 in schools; and during visits one participant was in bed, two in standers, 141 in wheelchairs, and 36 in adaptive chairs. All testing was carried out in a quiet room with normal indoor temperature.

Summary statistics including the mean RSA values across baseline and the five sensory domains and total RSA and RMSSD values across the PASMA testing for the entire CMC group, as well as CP and non-CP subgroups, are reported in Table 3. Controlling for sensory domain and temporal changes, the CP group demonstrated significantly higher RSA values relative to the non-CP group. Specifically, model results show that the probability of the CP group demonstrating an increase in RSA measures relative to the non-CP group was 99%, with the average increase observed being 0.56. See Table 4 for the remaining regression results. The total RSA and RMSSD values for all PASMA domains were also higher for the CP subgroup. A strong correlation of 0.81 between total RMSSD and RSA values was demonstrated (*p* < 0.05).

**Figure 1:**
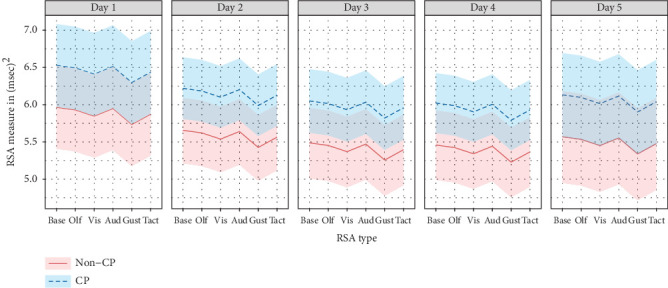
Estimated profile plot across sensory domains within any given day, stratified by cerebral palsy.

Figure 1 depicts the estimated profile trajectory plots of the RSA values for each participant across sensory domains within each visit having been stratified by whether the participant had CP or not. This plot depicts nearly similar estimated RSA values for the olfactory and auditory domains relative to baseline. It should be noted that the auditory domain demonstrated the highest RSA value for all CMC groups; however, Day 4 for the auditory domain demonstrated high missingness, which may have contributed to its insignificant finding. RSA values significantly differ from baseline for the gustatory domain.

**Figure 2:**
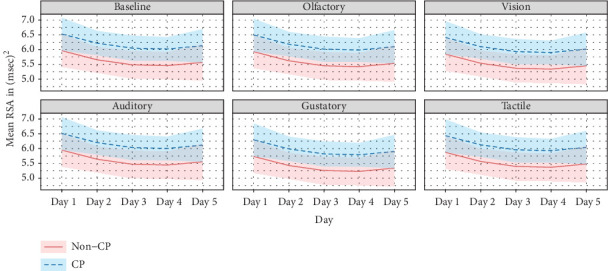
Estimated profile plot across time within any given sensory domain, stratified by cerebral palsy.

Figure 2 presents the estimated profile trajectory plots of the RSA values for each participant across the days within each modality with the same CP stratification. This plot indicates that across days there appears to be a learning curve in which participants' RSA values decrease over each day until Days 3 (olfactory, vision, and tactile) and 4 (auditory, gustatory) when values begin to improve. Missing data for Day 4 of auditory again may have skewed this result. If this data had not been missing, indications of learning for auditory would have likely occurred on Day 3. While the above differences are noted graphically, they are not statistically significant.

## 4. Discussion

Findings from this study's results provide preliminary RSA values for CMC that may allow for comparisons. Within this study's population of CMC, comparisons between children with CP and non-CP highlight that the CP group had greater RSA values, which are indicative of greater parasympathetic regulation. When compared with children having autism and those considered typically developing, CMC display reduced RSA, which provides an indication of less parasympathetic regulation. This study's RSA baseline value of 6.0 for CMC was lower compared to the reported values of 6.84 and 6.91 for children with autism and those typically developing, respectively [17, 33]. In 2019 and using the RMSSD variable, Katz-Leurer and Amichai [12] reported baseline values for children with CP across the five GMFCS levels. Their work included 24 children with CP classified as GMFCS level V who demonstrated a RMSSD value of 29. This study documented a value of 28 for the total CMC group, 32 for the subgroup of children with CP (19/20 being consider GMFCS level V), and 22 for the subgroup of children with non-CP. This comparison between study values provides an additional indication that collectively CMC may experience less parasympathetic regulation than children with CP. However, it should be noted that this study's CP subgroup displayed a RMSSD value of 32, which is higher than the 29 reported by Katz-Leurer and Amichai [12]. Explanations for this may lie in establishing better diagnostic capacity to differentiate children with more complex CP from those defined as CMC.

Across sensory domains and for both groups of CP and non-CP, there is evidence that gustatory experiences were associated with reduced parasympathetic regulation, as reflected by lower RSA. Although this study cannot identify causation, reasonable hypotheses may be drawn by examining participant factors. Of the 36 CMC enrolled in this study, 27 used gastrostomy and/or jejunostomy tube as a primary means for receiving nutrition. For these children, gustatory experiences (i.e., introduction of liquid and/or food to the mouth) are limited, so the likelihood that these experiences would interfere with parasympathetic regulation is reasonable. It should also be noted that HRV is modulated by inhalation and exhalation [34]. Gustatory experiences are linked to the skill of eating and/or feeding, which requires coordination between sucking, swallowing, and breathing (i.e., inhalation and exhalation). This coordination is often motorically and sensory challenging for CMC [35], so it should not be surprising that gustatory activities would reduce RSA. These gustatory indications should inform providers and caregivers serving CMC that even minor gustatory experiences may result in initiating a fight or flight experience. However, it is also important to remember that because a learning effect was observed across PASMA administrations, rather than fully eliminating gustatory opportunities, a stepwise gustatory progression may be more ideal. Gustatory experiences have been linked to pleasure and enhanced quality of life in adults and children with marked neurodisability [36, 37]. Therefore, by providing CMC with safe and monitored gustatory opportunities, quality of life may be enriched.

In contrast to gustatory stimulation, there is an indication that auditory experiences (e.g., conversation, music) may be favorable and protective for CMC. Although no significant difference was noted, which may be reflective of missing auditory data on Day 4 of PASMA administration, summary statistics indicate a consistent RSA change in a positive and possibly protective direction. Although further hypothesizing and study specific to CMC is needed, evidence exists that notes HRV improvements in the presence of and following auditory experiences [38, 39]. A systematic review conducted by Mojtabavi et al. [40] confirmed that music therapy was associated with improved HRV for both healthy individuals and those with various diagnoses including those with neurological and psychiatric disorders [40, 41]. In adults with severe cognitive impairment similar to CMC, exposure to music was associated with significant behavioral benefit [42]. Again, further research is needed, but evidence suggests that appropriate and pleasant auditory experiences and interventions may mediate RSA improvement in CMC.

Although a definition for distinguishing CMC exists and multiple classification systems (i.e., GMFCS, MACS, CFCS, and EDACS) are available for differentiating those with more severe CP within the population of CMC, Berry et al. [43] have pointed out that it often remains difficult for health care practitioners to clearly identify CMC. The inability to clearly classify and discriminate CMC from other pediatric populations may hinder proper CMC resource utilization and/or inappropriately redirect funding to non-CMC populations. In conjunction with the existing CMC definition and CP classification systems, Berry and colleagues have suggested the incorporation of functional abilities and caregiver assistance measurement tools, such as the Pediatric Evaluation Disability Inventory-Computerized Adapted Test (PEDI-CAT) [44] to improve CMC identification [43]. This study's investigators agree with that recommendation and would also like to suggest that HRV analysis may also provide an opportunity to better distinguish CMC. However, identification of devices that would be clinically friendly to use and easily record frequency and/or time domain components of HRV would be necessary [15].

While CMC are a heterogeneous group, there is some degree of commonality as the majority often experience pain, feeding difficulties, constipation, reflux, and musculoskeletal issues [45]. They also often lack the ability to effectively communicate, which interferes with the development of timely and preventative treatment approaches for the aforementioned symptoms. Since RSA is usually lowered when experiencing many of the above symptoms [46–50], using it as an assessment tool to alert providers and caregivers of a rising fight or flight condition may be beneficial. Again, if a reliable, responsive, and easily understood RSA monitor could be developed, caregivers might have a method for earlier recognition of symptoms that might allow for the delivery of protective interventions before symptom escalation. This same device also could be translated to a clinical environment that would allow pediatric health care practitioners to evaluate health status and responses more objectively in real time during assessment and treatment of CMC.

### 4.1. Study Limitations and Recommendations

One limitation of this study was the inability to capture the planned 5-min baseline (i.e., quiet and alert state), which was to establish a reference point that would allow comparisons to sensory domain values. With a mean baseline of 3 min and 3 s for the enrolled CMC participants, the planned 5-min observational baseline was not achieved. The primary reason for abandoning baseline data collection early was related to CMC participants becoming agitated or drifting to sleep, both of which would have had a negative impact on PASMA administration and RSA values. However, Berntson et al. have documented evidence that 3-min baselines are reliable for HRV interpretation [51]. For CMC who experience multiple impairments, a 3-min baseline may be more realistic and ideal.

Two methodological procedural recommendations were challenging to execute in our work. First, the study's exercise physiologist recommended establishing and recording a baseline between each of the PASMA's sensory domains, but this faced similar difficulties as those described with achieving a 5-min baseline. As such, the procedure to establish a baseline between each sensory domain was abandoned during data collection on the first participant by the study's PI. With the administration of the PASMA over 5 days, the collection of repeated sensory domain RSA values may have provided some degree of stability to values that could not be obtained through interdomain baselines. Secondly, while our study's HRV details did not comply with all RR intervals acquisition and HRV parameters as outlined by the guidelines provided by the Task Force of the European Society of Cardiology and the North American Society of Pacing and Electrophysiology [15, 52], our study did comply with proper guidelines for using the Porges method (CardioBatch) for the editing and analyses of data for HRV [53]. Study sample characteristics would not allow implementation of paced breathing. We would recommend better compliance with the HRV guidelines in the future, but we also recognize that complex impairments associated with CMC, such as a limited ability to pace breath, may always interfere with full guideline implementation. Our study was able to document the time of day, baseline activity/movement observations, and testing positions, which are known to affect HRV. For future studies involving CMC in which establishing stable HRV baseline(s) needs to be accomplished, researchers may wish to consider a 12–24-h data collection period that could be reviewed for baseline considerations.

The exploration of sensory responses occurred across five sensory domains, with each of those domains containing 3–10 questions for a total of 28 items. However, reported RSA values were reflective of a mean (based on 30-s epoch) across each of the sensory domains that collectively included all questions. This provides at best a broad interpretation of sensory responses. Much like Kolacz et al. [18] noted with their Brain Body Center Sensory Scale work in children with Fragile X Syndrome, advancing neurophysiological mechanistic approaches is warranted in CMC too.

A substantial study limitation that needs to be addressed or controlled during future studies involving CMC and HRV revolves around the administration of seizure medication. Evidence suggests that accurate interpretation of HRV data for individuals taking seizure medications, not seizures alone, may be problematic [54]. In this study, 15 of the 36 participants experienced seizures, but no documentation concerning medication administration was gathered. Future studies involving CMC should collect information concerning the administration of seizure medication and may wish to create exclusion criteria for participants prescribed seizure medication.

Lastly, it should be noted that study investigators were PASMA administrators who were not blinded during data collection or analyses. While investigators recognize this may have generated bias in the actual PASMA item scores, they do not believe the lack of blinding impacted the objective HRV measure collected from the reliable Firstbeat monitors. Replication reliability between these PASMA administrators was also not established. In future studies, PASMA reliability should be examined as a specific study aim, or if the PASMA is used as a measurement tool within a study, reliability should be established within and/or between test administrators.

## 5. Conclusion

In 2011 Cohen et al. [55] published an article entitled “*Children With Medical Complexity: An Emerging Population for Clinical and Research Initiatives”* to encourage the uptake of research involving CMC and families. While studies addressing models of care, utilization, and CMC medical curriculum exist, research efforts directly examining CMC remain reduced. Some of the reasons for this may include the heterogeneity that exist within samples of CMCs, the unpredictable nature of CMC's medical status, investigators' fear of causing harm to CMC, and a recognized disparity based on disability severity that limits research efforts [56]. Although findings from this study are not generalizable, this preliminary work provides insight that in spite of limitations, clinical research involving CMC can be successfully conducted. In the future, more neurophysiological approaches to study CMC should be considered.

In summary, findings from this study provide initial descriptive RSA values for CMC and allow for comparisons. Additional findings suggest that (1) HRV is a simple and valid method by which to assess the ANS of CMC that may inform assessment and treatment, (2) certain sensory stimulation may reduce or intensify RSA in CMC, and (3) given repetition, CMC experience a learning effect. Collectively, these findings integrated into a pediatric rehabilitation assessment and/or treatment may improve the ability to understand and support parasympathetic regulation in CMC and thereby enhance health, outcomes, and quality of life.

## Figures and Tables

**Table 1:** Example items from pediatric awareness and sensory motor assessment.

**Olfactory**

Generalized smell: Without touching the nose, place 1 of Sanford Mr. Sketch Scented Marker beneath the nostrils of the child for 30 s. Remove stimulus once response occurs. Complete for 4 markers. Allow a 30-s rest between scents. **Mint** 0 = No response or commonly observed motor response 2 = Localized response: nasal flaring, increased inhalation depth, increased inhalation rate, lip smacking, teeth grinding, chewing, tongue thrust, increased drooling, increased sucking, increased swallowing, smiling, pouting, grimacing, quieting or exaggeration of movement, appears to attend

**Vision**

**Visual response to threat with red pompom:** Pinching a small red pompom, draw a “+” within 1 in. of the right eye. Enter and exit from lateral aspect of right eye. Do not touch eyelashes or create a breeze. 0 = No response OR commonly observed motor response 2 = Increased blinking, look towards or away from stimulus, turn head toward or away from stimulus

**Auditory**

**IF no response from low intensity or conversational intensity hearing items** **High-intensity hearing** (approximately 115 dB): Have nurse, caregiver, or available individual stand behind child so he/she cannot see the individual. Approximately 12 in. from right ear, ask the individual to hold up iPad/iPod, which is set at maximum volume on the song “Happy Birthday” and play for 5 s. 0 = No response OR commonly observed motor response 2 = Turns eyes or moves head in direction of sound, quieting of common motor response, appears to attend

**Gustatory**

**Seeking taste:** Apply flavored lip-gloss to lips with a clean Q-tip. Observe for 30 s and score. Do not use lip-gloss with mint. (choose flavor from drop-down menu): cherry, strawberry, bubble gum 0 = No response OR commonly observed motor response 2 = Tongue seeks stimulus on lips

**Tactile**

**Monofilament:** Apply 4.31 (protective sensation) filament to locations. Apply pressure to facilitate bending of the filament and maintain bend for 1.5 seconds. Monitor response for 30 s. If no response, apply three consecutive repetitions of filament. Monitor response for 30 s. Right face Right hand Left face Left hand 0 = No response OR commonly observed motor response 2 = Smiling, frowning, grimacing, pouting, blinking, eyes open or close, vocalizations, withdrawal or seeking of stimuli, quieting of movement, appears to attend

**Table 2:** Participant characteristics.

	**Overall group**	**CP group**					**Non-CP group**	
			**III**	**IV**	**V**	**Gastronomy tube**	**Wheelchair users propelled by caregiver: 16**	**Gastronomy tube: 7**
		**GMFCS**	**0**	**1**	**19**	**20**		
		**MACS**	**3**	**2**	**15**			
		**CFCS**	**0**	**2**	**18**			
		**EDACS**	**1**	**4**	**15**			
*n* (%)	36 (100)	20 (55.56)					16 (44.44)	
Age (years)								
Mean	11	10					12	
Range	5–20	5–18					5–20	
Mode	10	6 and 10					16	
Gender								
Male	25	14					11	
Female	11	6					5	
Race								
American Indian	1	1					0	
Asian	1	1					0	
Native Hawaiian or Other Pacific Islander	0	0					0	
Black/African American	7	4					3	
White	19	9					10	
Unknown	8	5					3	
Ethnicity								
Hispanic/Latino	7	4					3	
Not Hispanic/Latino	27	16					13	
Co-occurring conditions								
Seizure	15	5					10	
Tracheostomy	4	1					3	

**Table 3:** Summary statistics.

**RSA values**	**Overall**	**Cerebral palsy**	**Noncerebral palsy**
*N* (%)	36 (100%)	20 (55.6%)	16 (44%)
Baseline RSA (mean (SD))	6 (1.63)	6.2 (1.72)	5.73 (1.46)
Olfactory RSA (mean (SD))	5.98 (1.7)	6.18 (1.86)	5.71 (1.45)
Vision RSA (mean (SD))	5.84 (1.56)	6.01 (1.8)	5.6 (1.11)
Auditory (mean (SD))	6.12 (1.86)	6.31 (2.21)	5.84 (1.2)
Gustatory (mean (SD))	5.86 (1.79)	6.14 (1.93)	5.34 (1.38)
Tactile (mean (SD))	5.84 (1.83)	6.16 (2.08)	5.4 (1.28)
Total RSA	5.90	6.15	5.56
Total RMSSD	27.87	31.54	21.98

**Table 4:** Summary of regression parameters.

	**Posterior mean (95% credible interval)**	**Posterior probability**
Intercept	6.26 (5.75, 6.75)	0
Olfactory	−0.01 (−0.19, 0.17)	0.55
Vision	−0.14 (−0.3, 0.03)	0.95
Auditory	0.004 (−0.23. 0.24)	0.49
Gustatory	−0.26 (−0.42, −0.1)	0.999
Tactile	−0.15 (−0.33, 0.04)	0.94
Cerebral Palsy	0.28 (0.04, 0.53)	0.01
Time	−0.38 (−0.93, 0.15)	0.92
Time [2]	0.07 (−0.06, 0.2)	0.14

## Data Availability

The data that support the findings of this study are available on request from the corresponding author. The data are not publicly available due to privacy or ethical restrictions.

## Appendix

### 1. Appendix 1: Statistical Model

Each observation *Y*_*ijk*_ is assumed to follow a multivariate normal distribution where *i* = 1, ⋯, *n* observations, *j* = 1, ⋯, 5 visits, and *k* = 1, ⋯, 6 modalities. This assumption allows for taking the correlation into account with mean vector ***μ***_*ij*_ and covariance matrix Σ, all of which are captured below.

 $$\begin{array}{c} Y_{ijk}∼\text{MVN}\left(\mu_{ij},\Sigma \right). \end{array}$$

Notably, the mean RSA for each *i*^th^ observation in the *j*^th^ visit for the *k*^th^ modality, denoted as *μ*_*ijk*_, corresponds to the linear structure of predictors, shown below. In addition, this includes a subject-level random effect *b*_*ik*_ for the repeated measures across visits.

 $$\begin{array}{c} \mu_{ijk}=\beta_{0}+\beta_{k}+\beta_{CP}\cdot {\text{CerebralPalsy}}_{i}+\beta_{\text{Time}}\cdot {\text{Time}}_{j}+\beta_{{\text{Time}}^{2}}\cdot {\text{Time}}_{j}^{2}+b_{ik}. \end{array}$$

The estimated RSA measure at baseline modality was captured from *β*_0_, as we assumed the estimate for baseline modality, *β*_1_, to be constant with a mean of 0 and SD of 1. This method provided the estimated differences in the RSA values from baseline for the remaining five modalities in addition to capturing the correlation between modalities. Each fixed effect aside from *β*_1_ follow a normal distribution, *β*_*p*_ ~ *N*(0, *σ*_*β*_^2^), where *p* denotes the*p*^th^ fixed effect. The hyperprior for *σ*_*β*_^2^ follows a gamma distribution, *σ*_*β*_^2^ ~ Gamma(2,0.5). In addition, *b*_*ik*_ represents a subject-level random effect for a given defect for the repeated measures across visits and assumes to be independent with zero mean and follows a normal distribution prior, *b*_*ik*_ ~ *N*(0, *σ*_*bk*_^2^), with a hyperprior for *σ*_*bk*_^2^ that follows a gamma distribution, *σ*_*bk*_^2^ ~ Gamma(2,0.5). Σ assumes a Wishart prior distribution, Σ ~ Wishart(**R**, *s*) with scale matrix **R** and degrees of freedom *s*. As we assumed there was little variability in the modalities, we set *s* = 8. Of note, this is larger than the number of random parameters, which is the number of modalities, 6. The larger degrees of freedom increased the information obtained from the Wishart prior.
